# Combination chemotherapy consisting of irinotecan, etoposide, and carboplatin for refractory or relapsed neuroblastoma

**DOI:** 10.1002/cam4.4529

**Published:** 2022-03-01

**Authors:** Masayuki Imaya, Hideki Muramatsu, Atsushi Narita, Ayako Yamamori, Manabu Wakamatsu, Taro Yoshida, Shunsuke Miwata, Kotaro Narita, Daisuke Ichikawa, Motoharu Hamada, Eri Nishikawa, Nozomu Kawashima, Nobuhiro Nishio, Seiji Kojima, Yoshiyuki Takahashi

**Affiliations:** ^1^ Department of Pediatrics Nagoya University Graduate School of Medicine Nagoya Japan; ^2^ Department of Advanced Medicine Center for Advanced Medicine and Clinical Research Nagoya University Hospital Nagoya Japan

**Keywords:** chemotherapy, neuroblastoma, pediatric cancer, polymorphisms

## Abstract

**Background:**

Patients with primary refractory and relapsed neuroblastoma have a poor prognosis since safe and effective chemotherapies for these patients are currently limited. The development of new chemotherapy regimens for these patients is imperative to improve survival outcomes.

**Methods:**

We retrospectively analyzed 40 patients with refractory (*n* = 36) or relapsed (*n* = 4) neuroblastoma who received irinotecan, etoposide, and carboplatin (IREC) as a second‐line treatment. We evaluated their therapeutic response and the toxicity of IREC. We also assessed the impact of *UGT1A1* gene polymorphisms, which are involved in irinotecan metabolism, on outcomes and toxicity.

**Results:**

A total of 112 cycles of IREC were administered to 40 patients with a median of 2 cycles per patient (range, 1–9). Six (15%) patients (*UGT1A1* wild‐type [*n* = 2] and heterozygous [*n* = 4]) showed objective responses, including partial response (*n* = 1), tumor shrinkage (*n* = 4), and improved findings on their MIBG scan (*n* = 1). Grade 4 neutropenia, grade 4 leukopenia, and grades 3–4 gastrointestinal toxicity were observed in 110 (98%), 88 (79%), and 3 (3%) cycles, respectively. There was no IREC‐related mortality. Patients with *UGT1A1* polymorphisms showed a higher frequency of grade 4 leukopenia, but these patients did not have increased treatment‐related mortality or non‐hematologic toxicity.

**Conclusions:**

IREC showed an objective response rate of 15% including 1 case with partial response. IREC was well tolerated regardless of *UGT1A1* genotype. This study suggests that IREC is a promising second‐line chemotherapy for refractory or relapsed neuroblastoma.

## Graphical abstract

We evaluated the safety and efficacy of a 3‐drug combination of irinotecan, etoposide, and carboplatin (IREC) in 40 patients with refractory and relapsed neuroblastoma. The results showed that IREC was safe to administer, and 15% of patients showed tumor shrinkage and improvement in imaging findings.

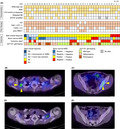

## 1 INTRODUCTION

Neuroblastoma is the most common extracranial solid tumor in children, representing approximately 8% of all childhood malignancies.^1^ The advent of multidisciplinary treatment approaches (including chemotherapy, surgery, radiotherapy, and high‐dose chemotherapy with autologous peripheral blood stem cell rescue) has helped improve the prognosis of patients with neuroblastoma, with overall survival rates increasing from 29% (1990–1994) to 50% (2007–2010).^2^ However, persistence or relapse of neuroblastoma occurs in >50% of patients, and they have unfavorable prognosis with long‐term EFS of 40%–50%^3^, ^4^ despite the development of various salvage therapies, including irinotecan alone^5^; irinotecan with temozolomide^6^; ifosfamide, carboplatin, and etoposide (ICE)^7^; topotecan and cyclophosphamide (TOPO‐CY)^8^; and the high‐dose ^131^I‐metaiodobenzylguanidine therapy.^9^ In recent years, although the anti‐GD2 immunotherapy has become widely incorporated into the treatment of high‐risk neuroblastoma, the response rate in relapsed or refractory neuroblastoma is unsatisfactory even when combined with chemotherapy.^10^, ^11^ Therefore, the development of new chemotherapeutic regimens is still imperative for patients with relapsed or refractory neuroblastoma.

A previous study reported outcomes after treatment with irinotecan, etoposide, and carboplatin (IREC) in 5 patients with neuroblastoma.^12^ As the best overall response, one relapsed patient achieved a complete response (CR) and the other four refractory patients achieved stable disease (SD). In that study, adverse effects of IREC, mild myelosuppression, and diarrhea, were minimal. Thus, IREC is a potentially valuable treatment for neuroblastoma, but it has not been subsequently studied in a larger cohort. In this study, we retrospectively assessed the response and safety profile of IREC treatment in 40 patients with refractory or relapsed neuroblastoma.

Irinotecan is metabolized in the liver and is converted into SN‐38, the active metabolite, which has an antitumor effect and has been further conjugated by UGT1A1. *UGT1A1* genotypes affect its UDP‐glucuronosyltransferase (UGT) activity and is one of the causes of the inter‐individual difference in the side effects of irinotecan.^13^ In the present study, we also summarized the impact of the *UGT1A1* genotyping on treatment response and toxicity to IREC treatment.

## 2 METHODS

### 2.1 Patients

We retrospectively analyzed data from patients with primary refractory or relapsed neuroblastoma who received IREC therapy at the Nagoya University Hospital, Nagoya, Japan, between October 2013 and March 2020. Primary refractory neuroblastoma was defined as inadequate response after ≥4 cycles of chemotherapy with the primary tumor with image‐defined risk factor scoring positive or with the presence of metastasis. They received a combination of salvage chemotherapy regimens selected from among IREC, ICE, TMZ+CPT11, and TOPO+CY, based on their treatment history and the possibility of residual drug sensitivity. Written informed consent for treatment with the IREC regimen was obtained from the patients or their parents before the start of IREC treatment. This study was approved by the ethics committee of the Nagoya University Graduate School of Medicine and was conducted in accordance with the principles of the Declaration of Helsinki. The data that support the findings of this study are available from the corresponding author upon reasonable request.

### 2.2 Treatment

IREC therapy consisted of the administration of irinotecan 100 mg/m^2^ (2‐h infusion), etoposide 100 mg/m^2^ (2‐h infusion), and carboplatin 80 mg/m^2^ (2‐h infusion) in three separate infusions on days 1 through 3. Trimethoprim–sulfamethoxazole and fluconazole were administered orally for infection prophylaxis. In patients with grade 4 neutropenia, prophylactic use of granulocyte colony‐stimulating factor (G‐CSF) was allowed. Blood transfusions were administered as appropriate with a target of hemoglobin >7 g/dl and platelet count >20 × 10^9^/L. Oral cefpodoxime was administered to prevent the development of diarrhea caused by irinotecan.^14^ We administered Hangeshashin‐to, a Kampo (Japanese herbal) medicine, to patients who developed diarrhea.^15^ IREC was administered at 4‐week intervals if bone marrow (BM) recovery (neutrophil count >0.5 × 10^9^/L and platelet count >100 × 10^9^/L) was achieved. Disease status was assessed by enhanced computed tomography (CT), magnetic resonance imaging, or Iodine 123 (^123^I) metaiodobenzylguanidine (MIBG) scintigraphy scans and BM aspirates from the bilateral posterior iliac spine. Response was assessed according to the International Neuroblastoma Response Criteria as the following: CR, meaning no evidence of disease; very good partial response, meaning that the volume of the primary mass was reduced by 90%–99%, with no evidence of metastasis (including normal MIBG) except for skeletal residua, and catecholamines were normal; partial response (PR), meaning a decrease of >50% in the size of the measurable tumor with ≤1 positive BM site; mixed response (MR), meaning a decrease of >50% of the size of any lesion with a decrease of <50% for any other lesion; no response (NR), meaning a decrease of <50% but an increase of <25% in the size of any lesion; and progressive disease (PD), a new lesion or an increase of >25% in the size of an existing lesion.^16^ SD was defined as encompassing MR and NR. Objective response (OR) was defined as CR, PR, and SD with tumor shrinkage or improved findings on the MIBG scan.

### 2.3 Follow‐up

We evaluated treatment response, survival outcomes, and toxicity using medical records. Follow‐up data available as of April 2020 were included in the analysis. The hematological and non‐hematological toxicity of IREC therapy were graded according to the National Cancer Institute Common Terminology Criteria for Adverse Events v.5.0.

### 2.4 UGT1A1 genotyping analysis

*UGT1A1* genotyping was performed using the Invader assay, a DNA analysis method that consists of a two‐step isothermal reaction using a structure‐specific flap endonuclease, as previously reported.^17^ On the basis of the results of testing for the *UGT1A1* *6 and *28 genetic polymorphisms, the patients were divided into three groups: wild‐type (−/−), heterozygous (*28/− and *6/−), and homozygous (*28/*28, *6/*6, and *28/*6).^18^

### 2.5 Minimal residual disease measurement

Total RNA was isolated from BM samples using the RNeasy Mini Kit (Qiagen); RNA concentration was evaluated by spectrophotometry. Reverse transcription (RT) was performed using a Thermoscript RT‐PCR system (Invitrogen) according to the manufacturer’s instructions. Real‐time quantitative RT‐PCRs (RQ‐PCRs) were performed on an ABI Prism 7000 Sequence Detection System (Applied Biosystems). Ready‐made primers and TaqMan probes (Assays‐on‐Demand Gene Expression Product) for glyceraldehyde‐3‐phosphate dehydrogenase (*GAPDH*: Hs99999905_m1), tyrosine hydroxylase (*TH*; Hs01002188_g1), and paired‐like homeobox 2B (*PHOX2B*: Hs00243679_m1) were purchased from Applied Biosystems. mRNA expression was quantified using TaqMan Universal PCR Master Mix II (Applied Biosystems, cat no. 4440040). PCRs were performed in a total volume of 15 μl, and the thermal reaction conditions were as follows: 50°C for 2 min, 95°C for 10 min, followed by 40 cycles of 95°C for 15 s, and 60°C for 1 min, for which fluorescence was detected using the StepOne Real‐Time PCR System (Applied Biosystems).^19^, ^20^

### 2.6 Statistical analysis

Overall survival (OS) was calculated using the Kaplan–Meier method from the first day of IREC administration to death or the last follow‐up visit. The cumulative incidence of relapse and non‐relapse mortality was calculated using Gray’s test. We used the Chi‐squared test to analyze categorical variables and the Mann–Whitney *U*‐test to analyze continuous variables. All statistical analyses were performed using EZR (Saitama Medical Center, Jichi Medical University), which is a graphical user interface for R (The R Foundation for Statistical Computing).^21^

## 3 RESULTS

### 3.1 Patient characteristics

**FIGURE 1:**
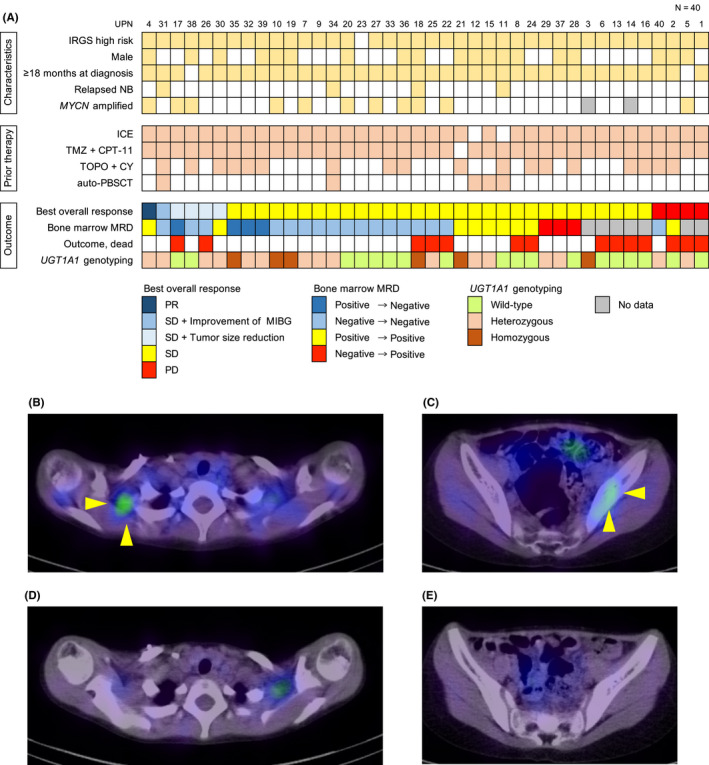
Clinical profiles of patients who received irinotecan, etoposide, and carboplatin (IREC) therapy. (A) Clinical profiles of the 40 patients in this study. Each column indicates 1 patient. (B–E) ^123^I‐MIBG scintigraphy in an 8‐year‐old boy with metastatic neuroblastoma (B, C) before and (D, E) after 3 courses of IREC therapy

Forty patients (19 males and 21 females) with primary refractory (*n* = 36) or relapsed (*n* = 4) neuroblastoma underwent IREC therapy (Figure 1A). The median age at diagnosis was 3.3 years (range, 1.2–10.0 years). Except for 1 patient with intermediate risk, the remaining 39 patients were classified as high risk on the basis of the International Neuroblastoma Risk Group classification. All 40 patients were previously treated with a median of 11 cycles (range, 6–21 cycles) of combination chemotherapy, including ICE (95%), irinotecan with TMZ (98%), and TOPO‐CY (45%) before IREC treatment. Five of the 40 patients received autologous peripheral blood stem cell transplantation (auto‐PBSCT) before IREC treatment: 3 patients received auto‐PBSCT and relapsed before IREC treatment. All patients had measurable lesions at the start of IREC. They received a median of 2 cycles (range, 1–9 cycles) of IREC treatment starting at a median of 0.8 years (range, 0.4–3.3 years) after diagnosis. According to the Lansky performance status scores, 2 patients scored 60 and 70, and the remaining 38 patients were normally active (score 90) at the start of IREC treatment. On the basis of *UGT1A1* genotyping analysis, the patients were divided into the wild‐type (*n* = 18), heterozygous (*n* = 16), and homozygous (*n* = 6) groups.

### 3.2 Treatment response

All patients were evaluated for treatment response, except for one patient who died because of tumor progression within 6 weeks of starting IREC treatment (Figure 1A). In total, six (15%) patients (*UGT1A1* wild‐type [*n* = 2] and heterozygous [*n* = 4]) showed an OR, including PR (*n* = 1), tumor shrinkage (*n* = 4), and improved findings on the MIBG scan (*n* = 1). PR was observed in one patient whose MIBG scintigraphy showed the disappearance of MIBG uptake in the bone metastases after the first cycle of IREC therapy and remained negative until the end of three treatment cycles (Figure 1B–E). Thirty‐five patients maintained SD after one to five cycles of IREC therapy, including four patients with reduced tumor size and one patient with improved MIBG scintigraphy results.

In 33 of 40 (83%) patients, BM minimal residual disease (MRD) was measured by RQ‐PCR for *TH* and *PHOX2B* at baseline and follow‐up. All were histopathologically negative for BM infiltration of neuroblastoma cells. At the start of IREC therapy, 13 patients were MRD‐positive, and in 4, MRD became negative after 1–3 cycles of treatment. Although 20 patients were MRD‐negative at the start of IREC treatment, in 3, MRD became positive after 1–3 cycles of IREC therapy.

### 3.3 Toxicity

All 40 patients received a median of 2 cycles (range: 1–9) of IREC therapy, and we assessed a total of 112 cycles for adverse events (Table 1). Twenty‐five cycles required dose reduction for renal damage, prolonged BM suppression, or schedule adjustments to meet the stem cell transplantation date. Grade 4 hematological toxicity included neutropenia in 110 cycles (98%), thrombocytopenia in 24 cycles (21%), and anemia in 1 cycle (1%). The median duration of grade 4 neutropenia was 8 days (range: 0–36) with the use of G‐CSF support. Of the 40 patients, 32 (80%) required red blood cell transfusion and 34 (85%) required platelet transfusion. The median number of transfusions for each cycle was 1 (range: 0–9) for red blood cells and 2 (range: 0–11) for platelets. Non‐hematological toxicity in grades 3–4 were as follows: three patients (3%) developed grade 3 diarrhea, but none of the patients developed grade 4 diarrhea. A fever of ≥38°C occurred in 47 cycles (42%), and febrile neutropenia (FN) occurred in 25 cycles (22%). Blood cultures were positive for bacteria in six patients; three of these patients required the removal of a catheter to control the infection. Renal and liver damage was transient and improved with conservative treatment. Patients with *UGT1A1* polymorphisms showed a higher frequency of grade 4 leukopenia compared to wild‐type patients (95% vs. 63%); however, there was no difference in the frequency of grades 3–4 diarrhea and FN.

**TABLE 1:** Adverse events

CTCAE version 5.0	Total cohort (112 courses, *n* = 40)	*UGT1A1* WT (56 courses, *n* = 18)	*UGT1A1* MT (56 courses, *n* = 22)	*p*‐value
Hematologic toxicity (grade 4)				
Anemia, *n* (%)	1 (0.9)	0	1 (1.8)	0.326
Leukopenia, *n* (%)	88 (78.6)	35 (62.5)	53 (94.6)	3.75 × 10^−5^
Neutropenia, *n* (%)	110 (98.2)	54 (96.4)	56 (100)	0.159
Thrombocytopenia, *n* (%)	24 (21.4)	12 (21.4)	12 (21.4)	1
Non‐hematologic toxicity (grades 3–4)				
FN, *n* (%)	25 (22.3)	9 (16.1)	16 (28.6)	0.115
Fever, *n* (%)	2 (1.8)	2 (3.6)	0	0.159
Diarrhea, *n* (%)	3 (2.7)	2 (3.6)	1 (1.8)	0.567
Nausea, *n* (%)	7 (6.3)	3 (5.4)	4 (7.1)	0.703
Vomiting, *n* (%)	5 (4.5)	4 (7.1)	1 (1.8)	0.174
CRBSI, *n* (%)	3 (2.7)	2 (3.6)	1 (1.8)	0.567
Meningitis, *n* (%)	1 (0.9)	0	1 (1.6)	0.326
AST elevation, *n* (%)	4 (3.6)	2 (3.6)	2 (3.6)	1
ALT elevation, *n* (%)	5 (4.5)	2 (1.8)	3 (5.4)	0.315
Amylase elevation, *n* (%)	4 (3.6)	2 (3.6)	2 (3.6)	1
Seizure, *n* (%)	1 (0.9)	0	1 (1.8)	0.326
Hypoxia, *n* (%)	1 (0.9)	0	1 (1.8)	0.326

Abbreviations: ALT, alanine aminotransferase; AST, aspartate aminotransferase; CRBSI, catheter‐related bloodstream infection; CTCAE, common terminology criteria for adverse events; FN, febrile neutropenia; MT, heterozygous and homozygous; WT, wild‐type.

### 3.4 Survival outcomes

After IREC therapy, 16 patients received a total of 24 stem cell transplantation procedures: 5 patients received autologous peripheral blood stem cell transplantation, 6 patients received CBT, and 5 patients received both. Treatment‐related death was observed in one patient (UPN13). This patient underwent autologous peripheral blood stem cell transplantation using a busulfan + melphalan regimen after three courses of IREC therapy; she died 6 months after transplantation owing to a late‐onset non‐infectious pulmonary complication.

**FIGURE 2:**
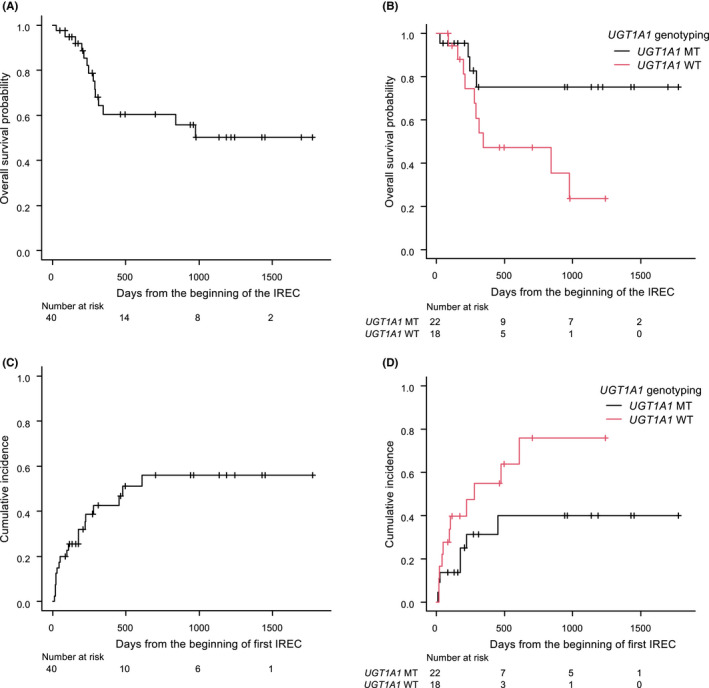
Overall survival and cumulative incidence of relapse or progressive disease. From the beginning of the first irinotecan, etoposide, and carboplatin (IREC) treatment, overall survival (OS) was calculated until death from any cause, and the cumulative incidence (CI) was calculated until relapse or progressive disease. The median duration of follow‐up for OS was 9.6 months (range: 0.9–58.3). (A) OS for all patients, (B) OS according to *UGT1A1* genotype, (C) CI for all patients; and (D) CI according to *UGT1A1* genotype

The 1‐year OS rate for the whole cohort was 60.5% (95% confidence interval, 0.406–0.755). There was no difference between primary refractory cases and relapsed cases with respect to the 1‐year OS rate (60.5% vs. 66.7%, *p* = 0.832). Patients with *UGT1A1* polymorphisms (both the heterozygous and homozygous groups) showed a better 1‐year OS than did wild‐type patients (75.2% vs. 47.3%, *p* = 0.038) (Figure 2A,B). There was no difference in the cumulative incidence of relapse in patients with and without *UGT1A1* polymorphisms (Figure 2C,D).

## 4 DISCUSSION

In this study, we evaluated the safety and efficacy of IREC therapy as a salvage chemotherapy regimen in 40 patients with primary refractory or relapsed neuroblastoma; 6 (15%) patients showed OR, including PR (*n* = 1), tumor shrinkage (*n* = 4), and improved findings in the MIBG scan (*n* = 1). These findings are consistent with those of a previous report by Inoue et al. in which one of five patients with refractory or relapsed neuroblastoma achieved CR with IREC therapy.^12^ The rate of CR + PR (3%) of IREC in our study tended to be lower than that of other salvage regimens for neuroblastoma (ICE, 18%^7^; TOPO‐CY, 46%^8^; irinotecan alone, 2%^5^; and irinotecan + temozolomide, 8%^6^) (Table S1). However, given that all 40 patients in this cohort were treated with multiple second‐line chemotherapy regimens, including ICE (95%), irinotecan with TMZ (98%), and TOPO‐CY (45%) prior to IREC, IREC therapy appears to be a promising treatment alternative for these patients.

Grade 4 hematological toxicity after IREC treatment was observed in 98% of patients; however, it was successfully managed with G‐CSF and transfusion therapy, and no deaths due to infection were recorded. The main non‐hematological toxicity was FN (22%); other non‐hematological toxicities ≥grade 3 were observed in only 10% of cases. The incidence of bloodstream infection (BSI) (6 of 40 patients and 5.4% of all cycles) was lower than that reported with ICE (26%)^7^ and was similar to that of irinotecan with temozolomide (3 of 47 patients).^6^ In addition to prophylactic oral cefpodoxime,^14^ supportive care with Hangeshashin‐to (Kampo medicine),^15^ may have contributed to the low incidence of high‐grade diarrhea in this cohort. Our results suggest that IREC is a relatively safe chemotherapy regimen for patients with refractory or relapsed neuroblastoma.

Irinotecan is hydrolyzed by carboxylesterase to its active metabolite SN‐38, which is then metabolized by UGT1A1 to the non‐toxic glucuronide SN‐38G. *UGT1A1* polymorphisms have been shown to decrease the metabolism of SN‐38 and are associated with a higher incidence of cytopenia and diarrhea, which are major side effects of irinotecan treatment.^22^ In contrast, an irinotecan‐containing chemotherapy regimen (carboplatin, dexamethasone, etoposide, and irinotecan [CDE‐11]) for adult diffuse large B‐cell lymphoma has been shown to improve prognosis in patients with the *UGT1A1* *6 polymorphism.^23^ Meanwhile, the VIT (oncovin, irinotecan, and temozolomide) regimen in 44 pediatric patients with relapsed/refractory solid tumors was associated with increased irinotecan‐related toxicity but tended to reduce the patient overall survival.^24^ In our study, patients with *UGT1A1* polymorphisms had an increased frequency of grade 4 leukopenia after IREC therapy but had no increase in FN or BSI compared with wild‐type patients. Meanwhile, four of the six patients with OR had a *UGT1A1* polymorphism. Although definitive conclusions cannot be described due to the small sample size, our results suggest that patients with *UGT1A1* polymorphism may have a better 1‐year OS compared to wild‐type patients.

There are several limitations to this study. First, this was a single‐center, retrospective study of a relatively small number of patients, and the treatments other than IREC were not standardized; a prospective study in a larger cohort should be conducted to more accurately evaluate the effects of IREC therapy. Second, this study evaluated the impact of *UGT1A1* polymorphisms on IREC therapy, but its prognostic value, especially impact on survival, should be cautiously interpreted, owing to the small number of patients and most of them received irinotecan‐containing chemotherapy other than IREC.

Third, patients who received anti‐GD2 antibody therapy, which is part of the current standard of care for patients with advanced‐stage neuroblastoma, were not included in the study. In the future, the safety of IREC therapy in patients previously treated with anti‐GD2 antibody therapy, or the safety of anti‐GD2 antibody therapy in patients previously treated with IREC therapy, will need to be evaluated. Fourth, while the clinical course clearly indicates that all patients have high‐risk neuroblastoma, most patients in this study were initially treated at other institutions; therefore, data on biological characteristics of the tumor are insufficient at the time of the initial diagnosis, which is necessary for proper risk classification, such as ploidy and 11q loss, other than *MYCN*.

In conclusion, the results of our study suggest that IREC is a promising second‐line chemotherapy for refractory or relapsed neuroblastoma and is well tolerated regardless of *UGT1A1* genotype. We hope to evaluate the efficacy of IREC in comparison with other salvage regimens in a large‐scale prospective study in the future.

## CONFLICTS OF INTEREST

There are no known conflicts of interest.

## AUTHOR CONTRIBUTIONS

Masayuki Imaya: Conception and design, collection of data, assembly of data, data analysis and interpretation, and writing, review and revision. Hideki Muramatsu: Conception and design, writing, review and revision. Atsushi Narita: Data analysis and interpretation, Ayako Yamamori, Manabu Wakamatsu, Taro Yoshida, Shunsuke Miwata, Kotaro Narita, Daisuke Ichikawa, Motoharu Hamada, Eri Nishikawa: Collection of data. Nozomu Kawashima, Nobuhiro Nishio, Seiji Kojima: Collection of data, supervision. Yoshiyuki Takahashi: Conception and design, supervision, review and revision. All authors were accountable for all aspects of the work and approved the final version of the article.

## ETHICAL APPROVAL

This study was approved by the ethics committee of the Nagoya University Graduate School of Medicine and was conducted in accordance with the principles of the Declaration of Helsinki. Written informed consent for treatment with the IREC regimen was obtained from the patients or their parents before the start of IREC treatment.

## Supporting information

Table‐S1

## Data Availability

The data that support the findings of this study are available from the corresponding author upon reasonable request.
