# Supplementary material for: Combination chemotherapy consisting of irinotecan, etoposide, and carboplatin for refractory or relapsed neuroblastoma
Source: Cancer Med. 2022 Mar 1;11(9):1956–64. doi: 10.1002/cam4.4529 (PMC9089216; doi:10.1002/cam4.4529)
Supplement: Supplementary file 1 — Table‐S1 [file CAM4-11-1956-s001.docx]

**Supplemental Table. Comparison of treatment response among second-line chemotherapy regimens for neuroblastoma**

| Regimen | No. of  patients | Best response, n (%) | | | | | | |
| --- | --- | --- | --- | --- | --- | --- | --- | --- |
|  |  | CR/  VGPR | PR | MR | OR | SD | PD | NE |
| ICE^1^ | 77 | 8 (10) | 6 (8) | 26 (34) | - | 23 (30) | 14 (18) | 0 |
| TOPO-CY^2^ | 13 | 0 | 6 (46) | 2 (15) | - | 0 | 5 (38) | 0 |
| Irinotecan alone^3^ | 44 | 1 (2) | 0 | - | - | 20 (45) | 13 (30) | 10 (23) |
| TMZ + Irinotecan^4^ | 36 | 2 (6) | 1 (3) | - | 9 (25) | 15 (42) | 9 (25) | 0 |
| IREC^5^ | 5 | 1 (20) | 0 | - | - | 4 (80) | 0 | 0 |
| IREC (this study) | 40 | 0 | 1 (3) | 0 | 5 (13) | 30 (75) | 4 (10) | 0 |

Abbreviations: CR, complete response; CY, cyclophosphamide; ICE, ifosfamide, carboplatin, and etoposide; IREC, irinotecan, etoposide, and carboplatin; MR, mixed response; NE, not evaluable; OR, objective response but less than PR; PD, progressive disease; PR, partial response; SD, stable disease; TMZ, temozolomide; TOPO, topotecan; VGPR, very good partial response.

References

1. Kushner BH, Modak S, Kramer K, Basu EM, Roberts SS, Cheung NKV. Ifosfamide, carboplatin, and etoposide for neuroblastoma: a high-dose salvage regimen and review of the literature. *Cancer*. 2013;119(3):665-671. doi:10.1002/cncr.27783
2. Saylors RL, Stine KC, Sullivan J, et al. Cyclophosphamide plus topotecan in children with recurrent or refractory solid tumors: a pediatric oncology group phase II study. *J Clin Oncol*. 2001;19(15):3463-3469. doi:10.1200/JCO.2001.19.15.3463
3. Kushner BH, Kramer K, Modak S, Cheung NK V. Five-day courses of irinotecan as palliative therapy for patients with neuroblastoma. *Cancer*. 2005;103(4):858-862. doi:10.1002/cncr.20846
4. Kushner BH, Kramer K, Modak S, Cheung NK V. Irinotecan plus temozolomide for relapsed or refractory neuroblastoma. *J Clin Oncol*. 2006;24(33):5271-5276. doi:10.1200/JCO.2006.06.7272
5. Inoue M, Yasui M, Sawada A, et al. Efficacy of combination chemotherapy consisting of irinotecan, etoposide, and carboplatin (IREC) in children with refractory solid tumors. *Japanese J Pediatr Oncol*. 2007;44(2):135-142.
